# Rates of Intentional and Unintentional Nonadherence to Peritoneal Dialysis Regimes and Associated Factors

**DOI:** 10.1371/journal.pone.0149784

**Published:** 2016-02-26

**Authors:** Zhen Li Yu, Vanessa Yin Woan Lee, Augustine Wee Cheng Kang, Sally Chan, Marjorie Foo, Choong Meng Chan, Konstadina Griva

**Affiliations:** 1 Department of Psychology, National University of Singapore, Singapore, Singapore; 2 Alice Lee Centre for Nursing Studies, National University Hospital, Singapore, Singapore; 3 Department of Renal Medicine, Singapore General Hospital, Singapore, Singapore; 4 Health Services Research Group, City University London, London, United Kingdom; Sao Paulo State University, BRAZIL

## Abstract

With increasing emphasis on expanding home-based dialysis, there is a need to understand adherence outcomes. This study set out to examine the prevalence and predictors of nonadherence among patients undergoing peritoneal dialysis. A cross sectional sample of 201 peritoneal dialysis patients recruited between 2010–2011 from Singapore General Hospital completed measures of quality of life, medication beliefs, self-efficacy and emotional distress. Nonadherence rates were high; 18% for dialysis, 46% for medication and 78% for diet. Intentional nonadherence was more common for dialysis (*p* = .03), whereas unintentional nonadherence was more common for medication (*p* = .002). Multivariate models indicated significant associations for higher education (intermediate vs low OR = 3.18, high vs low OR = 4.70), lower environment quality of life (OR = 0.79), dialysis self-efficacy (OR = 0.80) with dialysis nonadherence; higher education (OR = 2.22), self-care peritoneal dialysis (OR = 3.10), perceived necessity vs concerns over medication (OR = 0.90), self-efficacy (OR = 0.76) with nonadherence to medication. The odds for nonadherence to diet were higher among patients who were younger (OR = 0.96), of Chinese ethnicity (OR = 2.99) and those reporting better physical health (OR = 1.30) and lower self-efficacy (OR = 0.49). Nonadherence is common in peritoneal dialysis. Self-efficacy and beliefs about medication are promising targets for interventions designed to improve adherence.

## Introduction

Peritoneal dialysis (PD) is the most common form of home-based dialysis for patients with End Stage Renal Disease (ESRD) [1]. Whereas PD first procedures are still not widely adopted, there is increased interest in expanding utilization of PD to cater for the growth of patients requiring dialysis [2, 3] with urgent-start PD programmes gaining momentum in the Nephrology community [4, 5]. For patients on PD, performance of the required dialysis exchanges in effective and timely manner in conjunction with lifestyle changes in diet and medication intake are essential to ensure optimal clinical outcomes. Yet a recent review concluded that nonadherence rates in PD are alarmingly high, ranging from 2.6–53% for dialysis procedures, 3.9–85% for medication intake and 14.4–67% for diet/fluid restrictions [6]. Variation in observed rates may be explained by the lack of consensus on measures and criteria used to define nonadherence. Conceptual and methodological limitations related to use of composite unidimensional indices [7] or unreliable biomarkers [8] have also been noted.

As nonadherence can reduce treatment efficacy and increase risk for mortality, hospitalization and peritonitis [9, 10], more work is warranted to explore patterns of nonadherence behaviours and factors likely to be associated with nonadherence in this population. Identifying salient risk factors for nonadherence could facilitate early detection and inform targeted cognitive and behavioral interventions.

It is also clinically informative to distinguish intentional or unintentional nonadherence. In the case of intentional nonadherence, patients actively choose not to follow treatment recommendations. Such deliberate acts are mainly linked to patients’ motivation and beliefs about their treatment [11]. Unintentional adherence (e.g., forgetfulness, carelessness), on the other hand, does not involve deliberation and is generally associated with patients’ skills or ability to follow medical advice [11]. To date, only one study has explored intentional and unintentional nonadherence among patients on PD and reported greater rates of unintentional nonadherence relative to intentional nonadherence (20% vs 15% for medication and 26% vs 26% for diet) [12], in line with work in other chronic populations [13, 14].

Nonadherence may be influenced by many factors. Psychosocial factors such as patient beliefs about their treatment or self-efficacy expectancies or emotional distress, have been shown to be consistent and potentially modifiable predictors of adherence in patients with ESRD [15] but the data was inconclusive for PD patients as most of work has focused mainly on sociodemographic and clinical parameters [6]. Though limited, evidence on the role of quality of life (QoL), emotional distress and self-efficacy in adherence to PD regimes is beginning to emerge [6]. These factors have typically been studied in isolation rather than as cluster [12, 16] hence their joint or relative contribution is not known.

The aims of the present study were (a) to document overall, intentional and unintentional nonadherence among PD patients with regards to dialysis, medication and diet and (b) to identify the most salient factors associated with nonadherence to each of these treatment aspects among a range of demographical, clinical and psychosocial parameters including emotional distress, QoL and patient beliefs.

## Materials and Methods

### Participants

Participants were recruited from the Peritoneal Dialysis Center, Singapore General Hospital (SGH) between 2010 and 2011. This is the largest national PD center in Singapore [17] providing care to majority of PD patients in Singapore. All consecutive adult patients in the clinic who were aged 21 and over, had been on PD programme for a minimum of 3 months, and were able to communicate in English, Mandarin or Malay were eligible to participate. Patients with severe mental constraints or those only fluent in dialects were excluded.

Patients were invited to participate while awaiting consultations with nephrologists at the PD center. Of 263 approached, 21 were excluded due to stroke (*n* = 3), dementia (*n* = 10), hearing difficulty (*n* = 7) and hospitalization (*n* = 1). Of the remaining 242 eligible patients, 201 consented to the study (response rate = 83%). Reasons for declining participation included lack of interest (*n* = 36), lack of time (*n* = 4) and frailty (*n* = 1). Following written consent, participants either self-completed (n = 107) or were administered the questionnaires as a structured interview by language-competent research personnel (n = 94) as per their preference. The SingHealth Centralised Institutional Review Board approved all study procedures.

### Measures

Medical notes were reviewed to record serological data (e.g., serum potassium, serum phosphate levels), comorbidities and other relevant clinical or treatment details (e.g., primary cause of ESRD, PD modality, dialysis vintage etc.). The Charlson Comorbidity Index (CCI) was used to consolidate comorbidity burden, computed pursuant to the method described by Beddhu et al. [18].

The study questionnaire for participants included five sections: demographics, nonadherence, QoL, emotional distress and health beliefs.

**Demographic** data included items such as respondent’s age, gender, relationship status, race/ethnicity, employment status, educational level, and household monthly income.

**Nonadherence** was assessed using self-report and clinical markers. The nonadherence self-report scale was adapted from Horne and Weinman [19] and comprised 3 items in each category (i.e., dialysis, medication or diet) to measure overall (e.g., “How often do you follow this regime?”), intentional (e.g., “Some people decide to miss out a dose of their medication or adjust it to suit their own needs. Overall, how often do you do this?”) and unintentional nonadherence (e.g., “Some people forget to take their medicines. Overall, how often does this happen to you?”). All items were rated on a 5-point scale ranging from 1 (= never) to 5 (= very often), with higher scores indicating greater nonadherence. Responses were dichotomized to identify patients never nonadherent and those at least occasionally nonadherent (overall, intentional or unintentional). Nonadherence was defined as at least occasionally nonadherent (i.e., score > 1 for each measure) [20]. Two additional items, modified from the USRDS Dialysis Morbidity and Mortality Study, were added to assess if patients have skipped or shortened their PD sessions over the past 4 weeks (i.e., “During the past 4 weeks, how many times have you shortened your PD session by 10 minutes?” for automated peritoneal dialysis, APD; “During the last 4 weeks, how many times have you skipped/missed one of your PD sessions/exchanges?” for continuous ambulatory peritoneal dialysis, CAPD) [21].

To supplement self-report, three biochemical markers (i.e., serum potassium, phosphate and albumin levels) were used to estimate nonadherence to diet and medication. Levels off recommended clinical targets (i.e. phosphate > 1.78 mmol/L; potassium < 3.5 mmol/L or > 5.1 mmol/L) [22, 23] were deemed indicative of nonadherence. Albumin levels < 3.5 g/dL [24] are indicative of malnutrition and may hence reflect dietary nonadherence. These cut-off values were also confirmed by the key PD consultants in patient care as clinical targets for the patient population.

**QoL** was operationalized with the Kidney Disease Quality of Life Short Form (KDQOL-SF) [25] and World Health Organization Quality of Life Instrument, Short Form (WHOQOL-BREF) [26]. SF-36 in the original KDQOL-SF questionnaire was replaced by its shorter version, SF-12 so as to reduce burden of completion for respondents. Two summary scores, physical component summary score (PCS) and mental component summary score (MCS), were calculated as per scoring procedures [27]. The kidney disease specific part includes 31 items to measure disease specific QoL, encompassing 6 domains: symptoms, effects of kidney disease, burden of kidney disease, patient satisfaction, staff encouragement and social support. Scores in each domain range from 0 to 100 with higher scores signifying better QoL.

WHOQOL-BREF includes 26 items. The first 2 items assess patients' overall QoL/health, ranging from 1 to 5. The other 24 items evaluate four QoL domains: physical health, psychological health, social relations, and environment. Each domain score range from 4 to 20, with higher score signifying better QoL.

**Emotional distress** was measured with the Hospital Anxiety and Depression Scale (HADS) [28] and the Revised UCLA Loneliness Scale (R—UCLA) [29]. HADS is widely used to measure symptoms of anxiety and depression in medical patients [30], as it excludes somatic symptoms such as fatigue, anorexia, and weight loss that may be related to comorbidity and uremia [31]. It comprises two 7-item scales, one for anxiety (HADS-A) and one for depression (HADS-D). Scores range from 0–21 on each scale, with higher scores indicating higher symptoms of depression/anxiety. HADS-A or HADS-D ≥ 8 indicates the presence of clinically relevant anxiety or depressive affect [32]. The HADS has been linguistically validated in both Mandarin [33] and Malay [34], which is a paramount consideration for use in the local context.

R—UCLA includes 20 items. Patients responded to the questions on a 4-point Likert scale. The aggregate score (range = 20–80) was calculated, with higher scores indicating a higher degree of loneliness.

**Beliefs about medication** were measured using the validated Beliefs About Medicines Questionnaire (BMQ) [35]. The BMQ has two subscales of five items each, measuring patients’ beliefs about the necessity of prescribed medication (e.g., “Without my medicines I would be very ill.”), and their concerns about potential adverse consequences of taking the medication (e.g., “Having to take medicines worries me.”). Within the subscales, items were rated from 1 (= strongly disagree) to 5 (= strongly agree) and summed to obtain a total score ranging from 5 to 25. Higher scores indicate stronger beliefs. A necessity-concerns differential was calculated by subtracting patients’ concerns score from the necessity score, ranging from -20 to 20 [11]. This demonstrates a cost—benefit analysis whereby a positive value indicates that the perceived benefits outweighs the cost.

**Self-efficacy** referring to individual’s confidence in executing specific behaviours to produce desired outcomes [36] was assessed using the Self-Efficacy for Managing Chronic Disease Scale developed by Lorig et al. [37] and a PD specific self-efficacy scale [12]. The latter was deemed essential as self-efficacy is task specific. The additional 7 items in the PD specific self-efficacy scale were generated following literature review, consultations with renal health care providers and pilot in a previous study [12]. The items rate on a scale from 1 (= not at all confident) to 10 (= totally confident), assessing patient confidence to carry out specific PD treatment-related behaviours: dialysis exchanges (1 item), medication (2 items) and diet (4 items). Higher scores indicate higher self-efficacy.

### Data Analysis

Intentional and unintentional nonadherence rates were compared using Statistics Calculator, version 4.0 (Stat Pac). Other statistical analyses were performed with SPSS software, version 17.0. Descriptive statistics were performed to provide information on the characteristics of the total sample and adherence subgroups. Univariate two-sample *t* tests or chi square tests were initially used to examine the association of each demographic, clinical, and psychological factor with each of the dichotomized nonadherence indices. All significant at p<.05 were subsequently used in multivariate logistic regression models (using backward entry) to determine those independently associated with nonadherence indices. For all analyses, complete cases were included and statistical significance was defined by p<.05.

## Results

### Study Participants

Patients’ characteristics are presented in Table 1. Study sample (mean age = 58.9, SD = 12.6 years) was predominantly Chinese, female and included *n* = 86 (43%) patients on automated peritoneal dialysis (APD) and 115 (57%) patients on continuous ambulatory peritoneal dialysis (CAPD).

**Table 1:** Sociodemographic and clinical characteristics for total sample and as stratified by adherence status on diet, medication and diet.

Variable	Total Sample	Dialysis ADH	Dialysis Non ADH	*p*	MedicationADH	MedicationNon ADH	*p*	Diet ADH	Diet Non ADH	*p*
No. of patients	201	165 (82)	36 (18)		109 (54)	92 (46)		41 (22)^a^	142 (78)^a^	
Demographics										
Age	58.9 ± 12.6	59.4 ± 13.2	56.8 ± 9.1	.2	60.9 ± 12.3	56.6 ± 12.7	.02	63.9 ± 10.3	56.9 ± 12.9	.001
Male	90 (45)	69 (42)	21 (58)	.07	50 (46)	40 (43)	.7	16 (39)	69 (49)	.3
Married	146 (73)	118 (72)	28 (78)	.4	79 (72)	67 (73)	.9	32 (78)	100 (70)	.3
Chinese	151 (75)	123 (75)	28 (78)	.7	79 (72)	72 (78)	.3	25 (61)	109 (77)	.04
Employed	55 (27)	45 (27)	10 (28)	.9	25 (23)	30 (33)	.1	6 (15)	44 (31)	.04
Education level				.03			.02			.2
Low	78 (39)	71 (43)	7 (19)		52 (48)	26 (28)		19 (46)	52 (37)	
Intermediate	81 (40)	62 (38)	19 (53)		37 (34)	44 (48)		17 (41)	56 (39)	
High	42 (21)	32 (19)	10 (28)		20 (18)	22 (24)		5 (12)	34 (24)	
Household monthly income^b^				.2			.9			.5
S$ 0-S$ 2000	65 (43)	50 (42)	15 (47)		32 (41)	33 (46)		8 (30)	49 (45)	
S$ 2001-S$ 4000	48 (32)	35 (29)	13 (41)		27 (34)	21 (29)		12 (44)	33 (30)	
S$ 4001-S$ 6000	15 (10)	12 (10)	3 (9)		8 (10)	7 (10)		3 (11)	11 (10)	
S$ 6001-above	23 (15)	22 (18)	1 (3)		12 (15)	11 (15)		4 (15)	17 (15)	
Dialysis-related characteristics										
APD	86 (43)	71 (43)	15 (42)	.9	38 (35)	48 (52)	.01	14 (34)	66 (46)	.2
Self-care PD	141 (70)	111 (67)	30 (83)	.06	68 (62)	73 (79)	.009	28 (68)	105 (74)	.5
Primary cause of ESRD				.2			.02			.2
Diabetes	85 (42)	71 (43)	14 (39)		55 (50)	30 (33)		22 (54)	58 (41)	
Hypertension	41 (20)	34 (21)	7 (19)		24 (22)	17 (18)		8 (20)	30 (21)	
Glomerulonephritis	55 (27)	47 (28)	8 (22)		22 (20)	33 (36)		10 (24)	37 (26)	
Others	20 (10)	13 (8)	7 (19)		8 (7)	12 (13)		1 (2)	17 (12)	
Previous Modality				.4			.5			.7
Prior HD	109 (54)	92 (56)	17 (47)		57 (52)	52 (57)		23 (56)	74 (52)	
Duration of dialysis (months)	42.2 ± 38.8	41.2 ± 37.8	44.6 ± 43.5	.7	40.7 ± 37.3	43.9 ± 40.6	.6	43.0 ± 38.7	38.6 ± 33.6	.5
CCI	5.7 ± 2.0	5.7 ± 2.0	5.5 ± 1.7	.5	6.0 ± 1.8	5.3 ± 2.0	.01	6.2 ± 1.8	5.5 ± 2.0	.07
Diabetes	95 (47)	80 (49)	15 (42)	.5	60 (55)	35 (38)	.02	22 (54)	66 (47)	.4
Hypertension	192 (96)	159 (96)	33 (92)	.2	104 (95)	88 (96)	.9	39 (95)	137 (97)	.7
Cardiovascular Disease	82 (41)	66 (40)	16 (44)	.6	47 (43)	35 (38)	.5	16 (39)	59 (42)	.7
Creatinine (mmol/L)	0.85 ± 0.31	0.84 ± 0.31	0.89 ± 0.31	.4	0.83 ± 0.31	0.87 ± 0.30	.3	0.86 ± 0.30	0.86 ± 0.31	.9
Potassium (mmol/L)	4.1 ± 0.6	4.1 ± 0.6	3.9 ± 0.6	.04	4.1 ± 0.6	4.1 ± 0.6	.8	4.2 ± 0.6	4.0 ± 0.6	.3
Phosphate (mmol/L)	1.6 ± 0.5	1.6 ± 0.6	1.5 ± 0.4	.1	1.6 ± 0.5	1.7 ± 0.5	.08	1.6 ± 0.5	1.7 ± 0.6	.4
Albumin (g/dL)	2.9 ± 0.5	2.9 ± 0.5	2.8 ± 0.5	.6	2.9 ± 0.5	3.0 ± 0.6	.6	2.9 ± 0.4	3.0 ± 0.5	.3
Hemoglobin (g/dL)	10.8 ± 1.6	10.8 ± 1.6	10.8 ± 1.9	.9	10.8 ± 1.5	10.9 ± 1.7	.8	10.6 ± 1.3	10.8 ± 1.7	.6
Kt/V^c^	2.3 ± 0.9	2.3 ± 1.0	2.3 ± 0.5	.7	2.3 ± 0.8	2.4 ± 1.1	.3	2.6 ± 1.7	2.2 ± 0.6	.2

Categorical variables are described as number (percentage); Continuous variables are described as mean ± SD.

ADH, adherence; APD, automated peritoneal dialysis; HD, hemodialysis; CCI, modified Charlson Comorbidity Index; ESRD, end stage renal disease.

^a^*n* = 18 patients reported no dietary restrictions.

^b^*n* = 50 did not wish to answer the question.

^c^*n* = 5 patients Kt/V values not available.

Most of the patients were on self-care PD (*n* = 141, 70%) and *n* = 60 (30%) patients were on assisted PD (i.e., dependent on caregivers for performance of dialysis). The overall group had a moderate level of comorbidity (CCI value = 5.7 ± 2.0). Depressive and anxious affect was common with 59.7% and 41.3% respectively scoring above the cut-off for probable cases.

### Rates of Nonadherence

Table 2 displays rates of nonadherence. Based on self-report, overall nonadherence rates were high, with a rate of 18% for dialysis, 46% for medication and 78% for diet. Intentional nonadherence was more common than unintentional nonadherence for dialysis (*p* = .03, 28% vs. 19%), whereas the opposite was observed for medication (*p* = .002, 58% vs. 73%). Diet intentional and unintentional rates were similar (*p* = .08, 84% vs. 77%).

**Table 2:** Adherence results based on self-report and biochemical markers.

Nonadherence variable	N (%)
Dialysis nonadherence	
Overall	36 (18)
Intentional	56 (28)
Unintentional	39 (19)
Medication nonadherence	
Overall	92 (46)
Intentional	117 (58)
Unintentional	146 (73)
Diet nonadherence	
Overall	142 (78)
Intentional	152 (84)
Unintentional	141 (77)
Shortening PD sessions	29 (14)
Skipping PD sessions	20 (10)
Potassium < 3.5 mmol/l	35 (17)
Phosphate > 1.78 mmol/l	69 (34)
Albumin < 3.5 g/dl	173 (86)

Nonadherence behaviours with respect to diet and medication were largely co-occurring (ps<.001) with 67.2% and 52.2% reporting lapses both due to forgetfulness and deliberation for diet and for medication respectively. For dialysis exchanges 11% reported both intentional and unintentional lapses. In addition, 14% of the patients reported shortening their PD sessions and 10% of the patients reported skipping the PD sessions over the past 4 weeks.

The proportions of participants with biochemical markers off clinical targets were 17% for potassium (indicative of dietary nonadherence) and 34% for phosphate (nonadherence to diet and/or intake of phosphate binders). A total of 86% patients did not achieve target albumin levels, suggesting that most patients were not eating appropriately.

### Univariate Associations with Nonadherence Measures

In the univariate analyses, the following statistically significant associations were found (see Tables 1 and 3):

**Table 3:** Means and SDs for QOL and psychosocial parameters for total sample and as stratified by adherence status on diet, medication and diet.

Variable	Total Sample	Dialysis ADH	Dialysis Non ADH	*p*	Medication ADH	Medication Non ADH	*p*	Diet ADH	Diet Non ADH	*p*
KDQOL-SF										
SF-12 PCS	36.0 ± 8.8	36.1 ± 9.1	35.4 ± 7.5	.7	35.4 ± 9.4	36.6 ± 8.2	.3	35.2 ± 9.7	36.4 ± 8.6	.4
SF-12 MCS	43.7 ± 11.2	44.6 ± 11.1	39.8 ± 10.8	.02	43.8 ± 12.3	43.7 ± 9.7	.9	44.6 ± 13.0	43.4 ± 10.6	.6
Symptoms	69.4 ± 19.2	70.4 ± 19.1	65.0 ± 19.7	.1	68.9 ± 19.8	70.0 ± 18.6	.7	71.8 ± 19.3	68.2 ± 19.3	.3
Effects of kidney disease	66.9 ± 22.0	68.2 ± 21.6	60.8 ± 22.9	.06	67.7 ± 23.0	65.9 ± 20.7	.6	68.1 ± 26.8	66.6 ± 20.2	.7
Burden of kidney disease	32.2 ± 26.3	33.6 ± 27.0	26.2 ± 21.8	.1	33.2 ± 29.0	31.1 ± 22.8	.6	30.0 ± 28.0	33.2 ± 25.7	.5
Patient satisfaction	65.5 ± 22.0	67.6 ± 22.3	56.0 ± 17.9	.001	68.8 ± 23.0	61.6 ± 20.2	.02	74.0 ± 20.8	63.6 ± 20.3	.005
Staff encouragement	73.8 ± 29.2	74.3 ± 30.2	71.2 ± 24.1	.6	76.0 ± 29.2	71.1 ± 29.1	.2	71.6 ± 36.7	76.3 ± 26.0	.4
Social support	70.4 ± 21.2	71.5 ± 21.7	65.5 ± 18.3	.1	71.5 ± 21.2	69.2 ± 21.4	.4	70.1 ± 20.8	70.4 ± 21.3	.9
WHOQOL-BREF										
Overall QoL/Health	3.1 ± 0.9	3.1 ± 0.9	2.8 ± 0.9	.06	3.1 ± 0.9	3.1 ± 0.8	.8	3.0 ± 0.9	3.1 ± 0.8	.5
Physical health	11.8 ± 3.2	11.9 ± 3.3	11.5 ± 2.8	.5	11.6 ± 3.5	12.1 ± 2.9	.3	10.9 ± 3.5	12.1 ± 3.1	.04
Psychological health	12.9 ± 3.1	13.2 ± 3.0	11.5 ± 2.9	.002	13.1 ± 3.3	12.6 ± 2.8	.2	13.1 ± 3.3	12.8 ± 3.1	.6
Social relations	13.2 ± 3.2	13.5 ± 3.2	11.9 ± 2.9	.006	13.5 ± 3.4	12.7 ± 2.8	.06	13.7 ± 3.8	13.1 ± 3.0	.3
Environment	13.4 ± 2.5	13.8 ± 2.4	11.8 ± 2.6	<.001	13.9 ± 2.7	12.9 ± 2.2	.003	14.5 ± 2.7	13.1 ± 2.4	.003
HADS										
Depression	9.0 ± 4.6	8.6 ± 4.6	10.7 ± 4.4	.02	8.8 ± 4.9	9.2 ± 4.4	.6	7.9 ± 5.2	9.2 ± 4.4	.1
Anxiety	7.0 ± 5.1	6.5 ± 5.0	9.3 ± 5.3	.002	7.2 ± 5.4	6.7 ± 4.8	.5	5.8 ± 5.3	7.3 ± 5.1	.1
R—UCLA	35.9 ± 9.4	35.0 ± 8.8	40.4 ± 10.6	.001	35.8 ± 9.7	36.1 ± 9.0	.9	35.0 ± 8.4	36.0 ± 9.6	.5
BMQ										
Necessity	20.3 ± 3.9	N/A	N/A		21.2 ± 3.4	19.2 ± 4.3	<.001	N/A	N/A	
Concerns	16.1 ± 5.0	N/A	N/A		15.3 ± 5.3	17.0 ± 4.5	.01	N/A	N/A	
Necessity-concerns differential	4.2 ± 5.8	N/A	N/A		5.9 ± 5.6	2.2 ± 5.5	<.001	N/A	N/A	
Self-efficacy										
General	5.9 ± 2.0	5.9 ± 2.0	5.9 ± 2.1	.9	5.8 ± 2.1	6.0 ± 1.9	.5	5.5 ± 2.2	6.0 ± 1.9	.2
Dialysis	8.9 ± 1.7	9.0 ± 1.7	8.1 ± 1.8	.004	N/A	N/A		N/A	N/A	
Medication	7.8 ± 2.0	N/A	N/A		8.2 ± 1.8	7.2 ± 2.1	<.001	N/A	N/A	
Diet	7.2 ± 2.1	N/A	N/A		N/A	N/A		8.5 ± 1.5	6.9 ± 2.0	<.001

ADH, Adherence; KDQOL-SF, Kidney Disease Quality of Life Short Form; SF-12, 12-item Short-Form Health Survey; PCS, physical component summary score; MCS, mental component summary score; WHOQOL-BREF, World Health Organization Quality of Life Instrument Short Form; QoL, quality of life; HADS, Hospital Anxiety and Depression Scale; R—UCLA, Revised UCLA Loneliness Scale; BMQ, Beliefs about Medicines Questionnaire.

High educational level (tertiary or above), low dialysis self-efficacy beliefs, low QOL (i.e. satisfaction with care, environmental QOL, social relations, psychological, MCS), high emotional distress (depression, anxiety, loneliness) were associated with higher dialysis nonadherence. High educational level, low comorbid burden, nondiabetic, CAPD modality, self-care PD, glomerulonephritis as primary cause of ESRD, low self-efficacy, medication beliefs (high concern and low necessity), low QOL (i.e. satisfaction with care, environmental and social relations) were associated with higher medication nonadherence. Younger age, being employed, Chinese ethnicity, low self-efficacy, high medication concerns, low QOL in terms of satisfaction with care and environmental QOL, high QOL in terms of physical health were associated with higher diet nonadherence. None of the remaining sociodemographic and clinical parameters was significant.

### Multivariate Analyses

The multivariate models to explain nonadherence to dialysis, diet and medication are shown in Table 4.

**Table 4:** Summary of Logistic Regression Analyses to predict nonadherence.

Dependent variables	Significant predictors	Odds ratio	Confidence interval	*p*
Overall Dialysis Nonadherence	Education (intermediate vs low)	3.18	1.16–8.67	.02
	Education (high vs low)	4.70	1.46–15.11	.009
	WHOQOL-BREF environment	0.79	0.65–0.96	.02
	Dialysis self-efficacy	0.80	0.66–0.98	.03
Overall Medication Nonadherence	Education (intermediate vs low)	2.22	1.08–4.54	.03
	Self-care PD	3.10	1.43–6.73	.004
	BMQ necessity-concerns differential	0.90	0.85–0.96	.002
	Medication self-efficacy	0.76	0.64–0.92	.004
Overall Diet Nonadherence	Age	0.96	0.92–0.99	.03
	WHOQOL-BREF physical health	1.30	1.12–1.51	.001
	Diet self-efficacy	0.49	0.36–0.69	<.001

WHOQOL-BREF, World Health Organization Quality of Life Instrument Short Form; BMQ, Beliefs about Medicines Questionnaire.

Education, WHOQOL-BREF environment and dialysis self-efficacy were significant multivariate associates of overall nonadherence to dialysis exchanges, with odds of nonadhererence increasing for patients with intermediate or high education (vs. low education) (odds ratio [OR], 3.18; 95% confidence interval [CI], 1.16–8.67 and OR, 4.70; 95% CI, 1.46–15.11 respectively), and decreasing with higher environment QoL (OR, 0.79; 95% CI, 0.65–0.96) and dialysis self-efficacy (OR, 0.8; 95% CI, 0.66–0.98).

The multivariate model for medication indicated that the odds for overall nonadherence to medication increased with higher education (OR, 2.22; 95% CI, 1.08–4.54), and self-care PD (OR, 3.10; 95% CI, 1.43–6.73). Higher medication necessity-concerns differential score (OR, 0.90; 95% CI, 0.85–0.96) and higher medication self-efficacy beliefs (OR, 0.76; 95% CI, 0.64–0.92) associated with lower medication nonadherence.

Significant factors associated with diet overall nonadherence included age, ethnicity, WHOQOL-BREF physical health, and diet self-efficacy. Diet nonadherence were more likely in Chinese patients (OR, 2.99; 95% CI, 1.15–7.81), in patients with better physical health QoL (OR, 1.30; 95% CI, 1.12–1.51) and less common in older patients (OR, 0.96; 95% CI, 0.92–0.99) and patients with higher level of diet self-efficacy (OR, 0.49; 95% CI, 0.36–0.69).

## Discussion

This study examined the prevalence and factors associated with nonadherence in PD patients. Observed nonadherence rates were consistent with past research [6, 20]. A total of 18%, 46% and 78% for nonadherence dialysis, medication and diet. PD patients were found to exhibit both intentional and unintentional nonadherence behaviours. The corresponding figures for intentional nonadherence to dialysis, medication and diet were 28%, 58% and 84% and 19%, 73% and 77% for unintentional nonadherence respectively. Frequency of unintentional nonadherence was higher than intentional nonadherence for medication, whereas the converse was observed for dialysis exchanges.

Overall, diet appeared to be the most problematic/challenging aspect [20] with intentional or unintentional lapses co-occurring. The intricacy of renal diet, with guidelines often contrasting those of healthy eating may explain the difficulty in assimilating, recalling and implementing guidelines in lifestyle [38]. Cultural and social norms around social significance of food especially for the Chinese and the culture of eating out in local context are also very pertinent [39], and may explain the higher rates of diet nonadherence among Chinese participants.

It is also important to note that dialysis nonadherence albeit lower than nonadherence for diet or medication, was not negligible. Intentional deviations from PD exchanges whereby patients may shorten, omit exchanges or otherwise deviate from prescribed PD regimes were more frequently reported than lapses due to forgetfulness. PD patients have more leeway to intentionally modify some aspects of their treatment such as the starting time on cycler, duration or number/interval of exchanges, since they are not dependent on the fixed schedule in dialysis centers, where attendance and dialysis procedures are monitored by health care professionals [40]. At home, the flexible routines may potentiate lapses or modifications in dialytic procedures especially when negotiating other competing activities of lifestyle demands.

Nonadherence can be positively or negatively reinforced. Adherence lapses may not be associated with perceptible negative effects or may even be rewarding e.g. when associated with some gain, enjoyment or symptom/burden relief [40]. Skipping a CAPD exchange for instance could alleviate abdominal discomfort without any immediate side effects. Nonadherence to hemodialysis regimen and remaining sick had been found helpful for patients to gain attention from others and to resolve family problems [41]. Moreover, the asymptomatic nature of many conditions for which dialysis patients must take medication may also discourage adherence [42].

Several demographic, clinical, and psychological factors were found to be associated with nonadherence. Among these, negative beliefs are of particular importance as they are potentially modifiable. Low self-efficacy consistently predicted nonadherence across all treatment aspects There is overwhelming theoretical and empirical evidence on the role of self-efficacy in adherence across a range of patient populations [43–46]. Self-efficacy training has been found to be effective in reducing nonadherence in hemodialysis [47] and enhancing QOL in PD [48].

Beliefs regarding medications are also crucial for medication adherence, as amply demonstrated in previous work [49]. What is of note however is that in our study costs vs. benefits analysis appears to be more salient than the separate constructs of concerns or necessity beliefs. In other words, the stronger the concerns medicines relative to perceived need, the less adherent patients are likely to be. This highlights the need to reinforce importance and personal need for medication while eliciting/addressing patients’ medication concerns [50].

The effects of socioeconomic parameters indicate that younger patients and those with higher education, on self-care PD and with better physical health had higher risk of nonadherence. These effects may reflect the practical and logistic challenges in following treatment for patients who are more likely to be occupationally and socially active [51]. The rigorous treatment demands may conflict with other activities and busy lifestyle [42]. Fear of being marginalized by social network if treatment guidelines are followed [52] may also be an issue especially when illness/or dialysis status has not been disclosed.

The observed associations between environment QoL and dialysis nonadherence are also noteworthy. PD regimes impact upon patients’ physical/home environment as they command space for machine and storage of equipment, or may pose limitations to socialization/visitations at home through the rigid sterilization techniques [52]. Patients, who nevertheless rate highly their environment QoL, are more likely to perceive their physical and built environment as safe, attractive or with available/adequate resources to meet their needs/interests. This can bolster positive attitudes towards home-based dialysis, which have been found to be important in motivating patients to perform exchanges [40].

Study findings have implications for clinical care. These include the need to screen and recognize nonadherence and negative beliefs as well as implement strategies to address barriers. As patients are unlikely to voice nonadherence unprompted [53, 54], it is important for renal health care professionals to raise such issues and create an environment to discuss challenges around adherence. Support strategies should consider where lapses are intentional or not. It is common to suggest behavioral strategies such as timer-alarms and medication boxes to patients reporting nonadherence [14]. These strategies alone are not likely to be effective without considering patients’ beliefs (self-efficacy and beliefs about medications).

Cognitive strategies/self-management interventions used as adjunct to routine care to modify dysfunctional beliefs and bolster self-efficacy may be particularly useful [55]. Eliciting and addressing patients’ beliefs and promoting an improved relationship between patient and healthcare provider have previously been associated with better adherence [49]. Integration of telehealth technology can also reinforce rapport and act as continuing line of support for the PD patients who administering treatment at home. Interventions using simple phone calls have been proven to be effective in improving QoL, resolving patients’ concerns and reducing nonadherence among PD patients and patients with other chronic conditions [56, 57].

The main limitations of our study relate to the cross sectional design that precludes causal inferences, the relatively small sample size and large number of variables studied. Study findings should be interpreted cautiously as significance levels were not adjusted multiple comparisons, thereby inflating Type I error. As there limited work on adherence in PD, the strategy adopted in this study was to be exploratory and generate hypotheses for further research.

Another limitation is possible selection bias. Several patients who either defaulted their regular appointment or declined to participate might also all have been nonadherent, leading to an underestimation of nonadherence rates. Moreover measurement is limited by the use of patient self-report as primary indicator of adherence which is more prone to bias and the adaptation of the original adherence scale [19]. Direct measures of nonadherence such as inventory checks/delivery records or other proxy clinical markers related to hydration/fluid status (e.g. blood pressure, bio-impedance spectroscopy) were not obtained or were not available during study window. Use of self-report was however unavoidable in soliciting information on intentional and unintentional non-adherence. It is of note that the significant associations between self-report and clinical proxy measures (i.e., potassium levels) allowed some confidence that biases were mitigated.

Furthermore, in light of the task-specific nature of any self-efficacy measurement we chose to develop a PD specific scale for the purposes of the study to measure ability to perform specific health/treatment related tasks. Although internal reliability coefficients were high, findings regarding nonadherence and self-efficacy should be interpreted with caution until replication.

In conclusion, this study assessed cognitions, emotional distress, QoL and nonadherence in a representative cohort of PD patients. High rates of nonadherence were observed for all three aspects of the medical regimen, i.e., dialysis, medication and diet, with dietary nonadherence being the most remarkable. Intentional nonadherence occurred more commonly than unintentional nonadherence for dialysis, whereas the opposite was observed for medication. More importantly, this study identified important determinants of nonadherence. These significant findings have important implications for the successful management of PD programme. Efforts aimed at improving patients’ communication with medical providers and addressing patients’ concerns about therapy or doubts about their own self-management capability are very likely to be effective in terms of reducing nonadherence and maximizing treatment effects in PD populations.
